# Retreatment and Reactivation Rates Following Bevacizumab, Ranibizumab, Aflibercept, and Laser for Retinopathy of Prematurity: A Systematic Review and Meta-Analysis

**DOI:** 10.7759/cureus.91652

**Published:** 2025-09-05

**Authors:** Arwa Alghamdi, Faisal Alasmari, Tala Aletani, Suzana Ezzi, Sereen Alharbi, Turki F Alasmari, Husain Alalgum, Muhannad Bin Sawad, Karim Talaat

**Affiliations:** 1 College of Medicine, King Saud Bin Abdulaziz University for Health Sciences, Jeddah, SAU; 2 Department of Vitreoretinal Surgery, Ophthalmology, King Saud Bin Abdulaziz University for Health Sciences, Jeddah, SAU

**Keywords:** antivascular endothelial growth factor, anti-vegf, laser therapy, retina, retinopathy of prematurity

## Abstract

Retinopathy of prematurity (ROP) is a vasoproliferative retinal disorder that affects preterm newborns. Although laser therapy has been the standard of care for ROP for decades, the use of anti-VEGF agents for ROP has recently become widespread. This systematic review and meta-analysis measures and compares the retreatment and reactivation rates following laser therapy versus anti-vascular endothelial growth factor (VEGF) agents and laser for ROP.

We searched Medline, Scopus, Clinical Trial, and Cochrane Library databases for all randomized controlled trials (RCTs) that have used bevacizumab, ranibizumab, aflibercept, or laser therapy for ROP. We assessed studies for risk of bias by the Critical Appraisal Skills Programme (CASP) criteria. Review Manager (RevMan) (2014) Version 5.3 was utilized to carry out the meta-analysis for our study.

Five studies were included in this review. The meta-analysis has revealed that laser treatment is associated with a significantly lower risk of retreatment than anti-VEGF medications (RR = 2.11, 95% CI 1.46-3.05, p < 0.0001, I² = 92%). In the subgroup analysis conducted based on the type of anti-VEGF medication used, studies using Aflibercept and Ranibizumab reported higher retreatment rates than studies with Bevacizumab. Similarly, there was a significant difference in terms of the reactivation rates, with patients who underwent laser therapy exhibiting lower reactivation rates than the anti-VEGF groups (RR = 2.00, 95% CI: 1.47 to 2.72, p < 0.0001, I² = 93%). The subgroup analysis comparing types of anti-VEGF agents has also displayed both ranibizumab and aflibercept to have higher reactivation rates than bevacizumab.

This meta-analysis demonstrates that laser therapy generally presents a notably reduced risk of retreatment and reactivation compared to ranibizumab and aflibercept, in line with prior research. Conversely, the study reveals that bevacizumab outperforms laser therapy, exhibiting lower rates of retreatment and reactivation.

## Introduction and background

Retinopathy of prematurity (ROP), formerly known as retrolental fibroplasia, is a vasoproliferative retinal disorder exclusive to preterm newborns [1]. Since retinal vascularization typically begins around the 12th week of gestation and concludes between 36 and 40 gestational weeks, premature infants often exhibit incomplete retinal vascularization at birth [1]. While spontaneous resolution is possible, untreated cases may progress to more severe conditions [1]. ROP is characterized by two phases, with the initial phase spanning from birth to 30-34 weeks of age [2]. During this phase, retinal vascularization decreases, largely due to elevated oxygen exposure from the external environment [2]. The relative hyperoxia of the extrauterine environment, along with supplemental oxygen often administered to premature infants, primarily drives this process [2].

However, beyond 30-34 weeks of age, as the retina's metabolic demands rise and vascularity decreases, the second phase ensues [3]. This phase initiates the growth of new vessels between the vascularized and avascularized regions of the retina as an adaptive response [3]. Nonetheless, this hypoxia-induced retinal neovascularization is pathological and can lead to fibrous scarring, potentially causing retinal detachment and blindness [2].

Elevated levels of vascular endothelial growth factor (VEGF) characterize this phase, as VEGF plays a pivotal role in vasculogenesis and angiogenesis [4]. Consequently, intravitreal injections of anti-VEGF agents have emerged as a therapeutic strategy for ROP [4]. Cryotherapy was historically the mainstay intervention for ROP, succeeded by laser therapy in the 1990s [3,5]. However, both methods of ablation have been associated with destructive effects, including high myopia, visual field loss, and retinal damage in some cases [3,6]. Conversely, studies have indicated that anti-VEGF treatments for ROP yield better refractive outcomes, fewer complications, and reduced reactivation rates [7-9].

Nevertheless, comprehensive evidence comparing the retreatment and reactivation rates of anti-VEGF therapy versus laser therapy for ROP remains relatively sparse and scattered. Thus, this paper aims to review eligible randomized controlled trials (RCTs) assessing and comparing the efficacy of anti-VEGF agents versus laser therapy for ROP, specifically examining the risk of disease reactivation necessitating retreatment. This article was previously posted as a preprint to ResearchGate in September 2023.

Methods

Registration

This study was registered on PROSPERO under the registration ID CRD42022328304.

Eligibility Criteria

All RCTs that evaluated the use of anti-VEGF agents and laser therapy for retinopathy of prematurity were deemed eligible for inclusion in this systematic review. Included population (P) was preterm infants diagnosed with treatment-requiring retinopathy of prematurity (ROP), regardless of gestational age, birth weight, or ROP zone; intervention (I) was intravitreal administration of anti-VEGF agents, including bevacizumab, ranibizumab, or aflibercept; comparator (C) was conventional laser photocoagulation therapy; and outcomes (O) were primary outcomes included retreatment rates and reactivation rates following initial therapy, and the study design (S) was only randomized controlled trials (RCTs).

Study Identification

We searched Medline, Scopus, Clinical Trial, and Cochrane Library databases for all related articles up to 2023 using the MeSH terms (or keywords when applicable) “anti-vascular endothelial growth factor” OR “anti-VEGF” OR “bevacizumab” OR “ranibizumab” OR “aflibercept” AND “laser” AND “retinopathy of prematurity” OR “ROP.” Two authors independently screened the articles based on titles and abstracts through Rayyan software, resolving discrepancies through consensus or consultation with a third independent reviewer. Following this initial screening, two reviewers separately assessed the full text of the selected articles to confirm eligibility, again resolving any discrepancies through consensus or consultation with a third reviewer.

Data Extraction

Two independent reviewers gathered data about the baseline characteristics and outcome measures of the studies included in our analysis. For each study, we collected information on population demographics (such as gestational age and birthweight), treatment methods, the number of eyes experiencing recurrence or necessitating retreatment, and the interval between initial and subsequent treatments. To prevent duplication of data in cases involving overlapping patient populations across studies, we prioritized the most comprehensive dataset. Given the specific focus of our study on ROP retreatment and reactivation rates, we did not extend our analysis to include comparisons of adverse effects such as myopia or diminished visual acuity, as these aspects fell outside the scope of our systematic review and meta-analysis.

Data Analysis

For conducting the meta-analysis in our study, we employed Review Manager (RevMan) Version 5.3 (2014). A confidence level of 95% was set, and statistical significance was determined with a p-value of less than or equal to 0.05. The outcomes were visualized using forest plots, and risk ratios (RR) were calculated for each of the variables under evaluation.

Evaluation of Study Quality

We applied the Critical Appraisal Skills Programme (CASP) criteria for randomized controlled trials [10] to evaluate the included studies for potential bias. The criteria focused on three main domains: study design, research methodology, and study results.

## Review

Results

Literature Search

**Figure 1:**
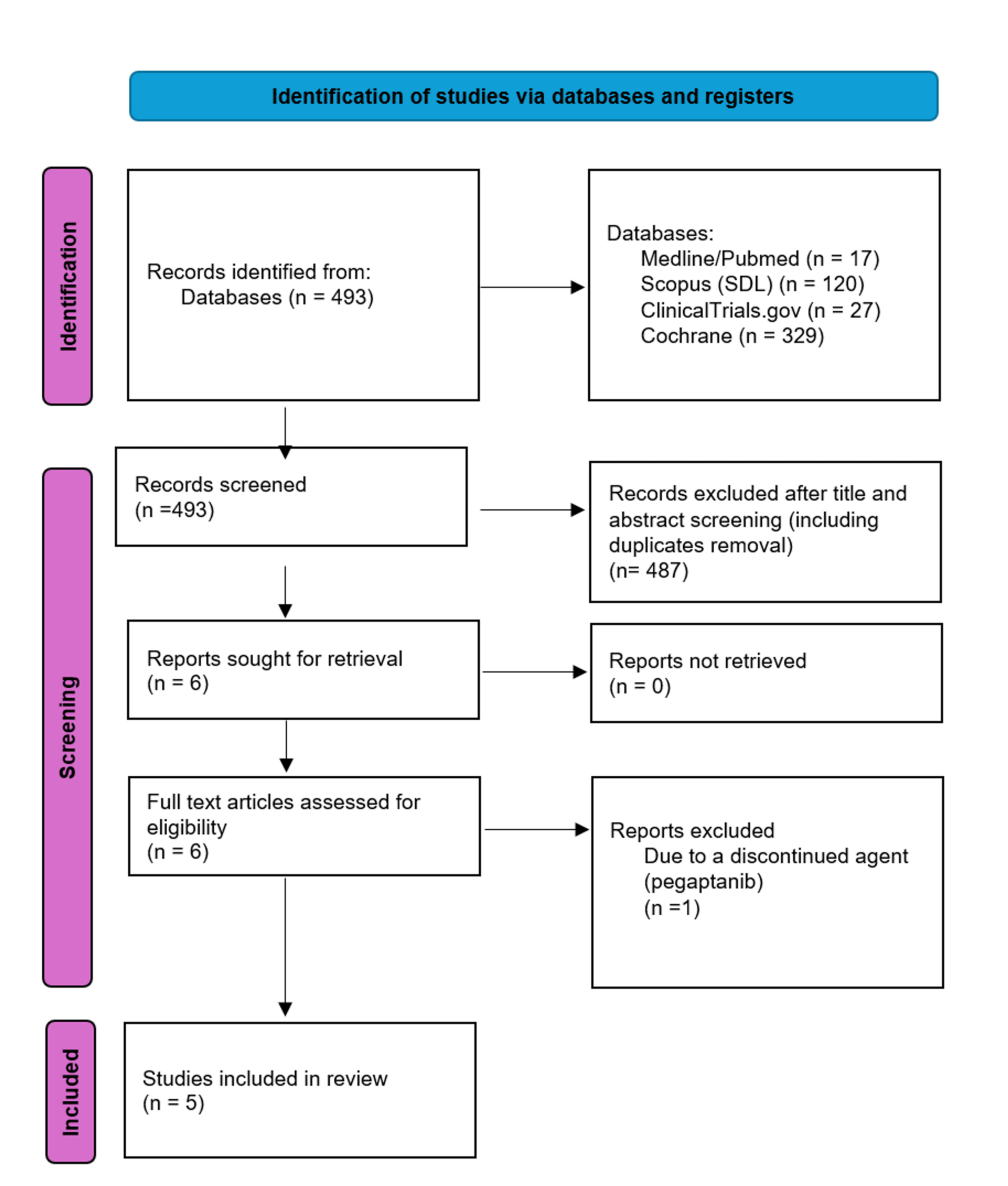
PRISMA flowchart for search and screening process.

A total of 493 studies underwent screening by two authors based on their titles and abstracts, utilizing specified inclusion and exclusion criteria to assess eligibility. Among these articles, 487 were excluded, including duplicates, leaving six potential studies. Following the assessment of these six papers by two reviewers, with discrepancies resolved through consensus or consultation with a third reviewer, one additional study was excluded due to the discontinuation of the agent pegaptanib (known as Macugen), rendering it no longer available. Consequently, five studies were deemed eligible for inclusion in this systematic review (Figure 1). Among the five included studies, two trials compared ranibizumab to laser therapy, two compared bevacizumab to laser therapy, and one compared aflibercept to laser therapy (Table 1). These five studies encompassed 191 preterm infants diagnosed with ROP in Zone I and 418 infants with ROP in Zone II; further details are provided in Table 2.

**Table 1:** Baseline characteristics of all studies included in the analysis. IVA: intravitreal aflibercept, IVB: intravitreal bevacizumab, IVR: intravitreal ranibizumab, N/A: not available, RCT: randomized controlled trial.

Study	Treatment Type				Study Design	No. of Patients	Total No. of Eyes	No. of Eyes Receiving Initial Treatment				No. of Eyes Requiring Retreatment				Mean Time Between Initial and 2nd Treatment		
	Bevacizu-mab	Ranibiz-umab	Afliberce-pt	Laser				IVB	IVR	IVA	Laser	IVB	IVR	IVA	Laser	IVB	IVR	IVA
Stahl et al. [11]	N/A	IVR (0.2mg and 0.1mg)	N/A	Laser	RCT	225	450	0	0.2mg 148 eyes 0.1mg 154 eyes	0	148	N/A	24 eyes in 0.2 24 eyes in 0.1	N/A	1	N/A	0.2mg: 55 days (range 29−111) 0.1mg: 57 days (30−128)	N/A
Karkhaneh et al. [12]	IVB (0.625mg/0.025ml)	N/A	N/A	Laser	RCT	79	158	86	0	0	72	9	N/A	N/A	1	5 ±1.66 weeks	N/A	N/A
Zhang et al. [13]	N/A	IVR (0.3mg/0.03ml)	N/A	Laser	RCT	50	100	0	50	0	50	N/A	26	N/A	2	N/A	12.62 weeks ± 7.93	N/A
Mintz-Hittner et al. [14]	IVB (0.625mg/0.025ml)	N/A	N/A	Laser	RCT	143	286	66	0	0	68	6	N/A	N/A	32	8.6 ± 19.2 for zone I 14.4 ± 0.8 for zone II	N/A	N/A
Stahl et al. [15]	N/A	N/A	IVA (0.4mg)	Laser	RCT	113	218	0	0	146	76	N/A	N/A	26	5	N/A	N/A	37 weeks

**Table 2:** The number of infants in each zone in all included trials. Zone 1: The most posterior zone, representing the central retina (macula and peripapillary area). Zone 2: Extends from the edge of zone 1 to the nasal or a serrata, including the mid-peripheral retina.

Author	Number of treated infants with zone I and zone II
Stahl et al. [11]	86 infants with zone I and 138 infants with zone II
Karkhaneh et al. [12]	79 infants with zone II
Zhang et al. [13]	50 infants with zone II
Mintz-Hittner et al. [14]	64 infants with zone I and 79 infants with zone II
Stahl et al. [15]	41 infants with zone I and 72 infants with zone II

Overall, these studies involved 1064 eyes of 610 infants, with 352 eyes receiving ranibizumab, 152 eyes receiving bevacizumab, 146 eyes receiving aflibercept, and 414 eyes receiving laser therapy. It is worth noting that various definitions of ROP reactivation, retreatment, and treatment failure were observed across the literature. Thus, the specific definition used in each study is outlined in Table 3. While several studies mentioned "recurrence of ROP activity," it's important to highlight that the third edition of the International Classification of Retinopathy of Prematurity recommends the use of the term "reactivation" to denote the recurrence of acute-phase features.

**Table 3:** The definitions of treatment failure, reactivation, and retreatment in each of the included studies.

Author	Treatment failure	Reactivation	Retreatment
Stahl et al. [11]	Any laser photocoagulation treatment received 11 days after baseline	Any ROP requiring treatment after baseline treatment	Receiving the same modality as the one administered at baseline
Karkhaneh et al. [12]	ROP persistence or recurrence	New extraretinal fibrovascular proliferation with the arrest of the anterior progression of the retinal vasculature during the follow-up period	Receiving the same modality as the one administered at baseline
Zhang et al. [13]	Not mentioned	Any of the following: recurrent plus disease, recurrent neovascularization, or reformation of ridge despite treatment	Receiving the same modality as the one administered at baseline
Mintz-Hittner et al. [14]	The recurrence of neovascularization in one or both eyes arising from the retinal vessels and requiring retreatment by 54 weeks’ postmenstrual age (with ascertainment performed between 50 and 70 weeks)	Neovascularization in one or both eyes arising from the retinal vessel	Receiving the same modality as the one administered at baseline
Stahl et al. [15]	Retreatment did not constitute treatment failure	Presence of initial disease improvement to a stage not requiring treatment, and subsequent worsening to require treatment again	Receiving the same modality as the one administered at baseline

Assessment of bias

**Figure 2:**
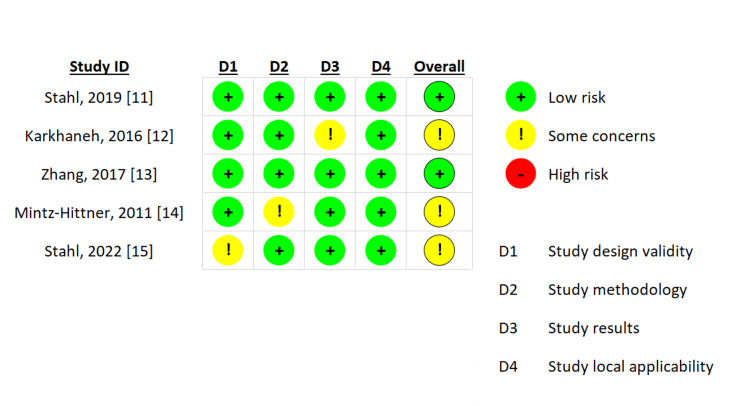
Summary of risk of bias assessment of included studies by critical appraisal skills programme (CASP) tool.

The comprehensive analysis conducted for this systematic review and meta-analysis demonstrated minimal risk of bias overall. The majority of RCTs included in this paper were found to have a low risk of bias, as depicted in Figure 2. Some studies raised concerns regarding bias due to inadequate or unclear reporting of masking procedures, intention-to-treat analyses, matching of participants' baseline characteristics, and/or precision of the intervention estimate. Given the nature of the interventions and the population studied (infants), participant blinding was often impractical in most trials. Consequently, it was deemed an unlikely source of bias.

Heterogeneity

While all articles incorporated in this systematic review were trials of RCTs, the variability in the types of anti-VEGF medications and doses, coupled with the limited number of available trials, has contributed to the diversity observed among the included articles. Specifically, concerning retreatment, significant heterogeneity was noted among studies (p<0.00001, I^2=94.1%), and a similar observation was made regarding reactivation, indicating substantial total heterogeneity (p<0.00001, I^2=95.1%).

Retreatment of Anti-VEGF vs. Laser Therapy

**Figure 3:**
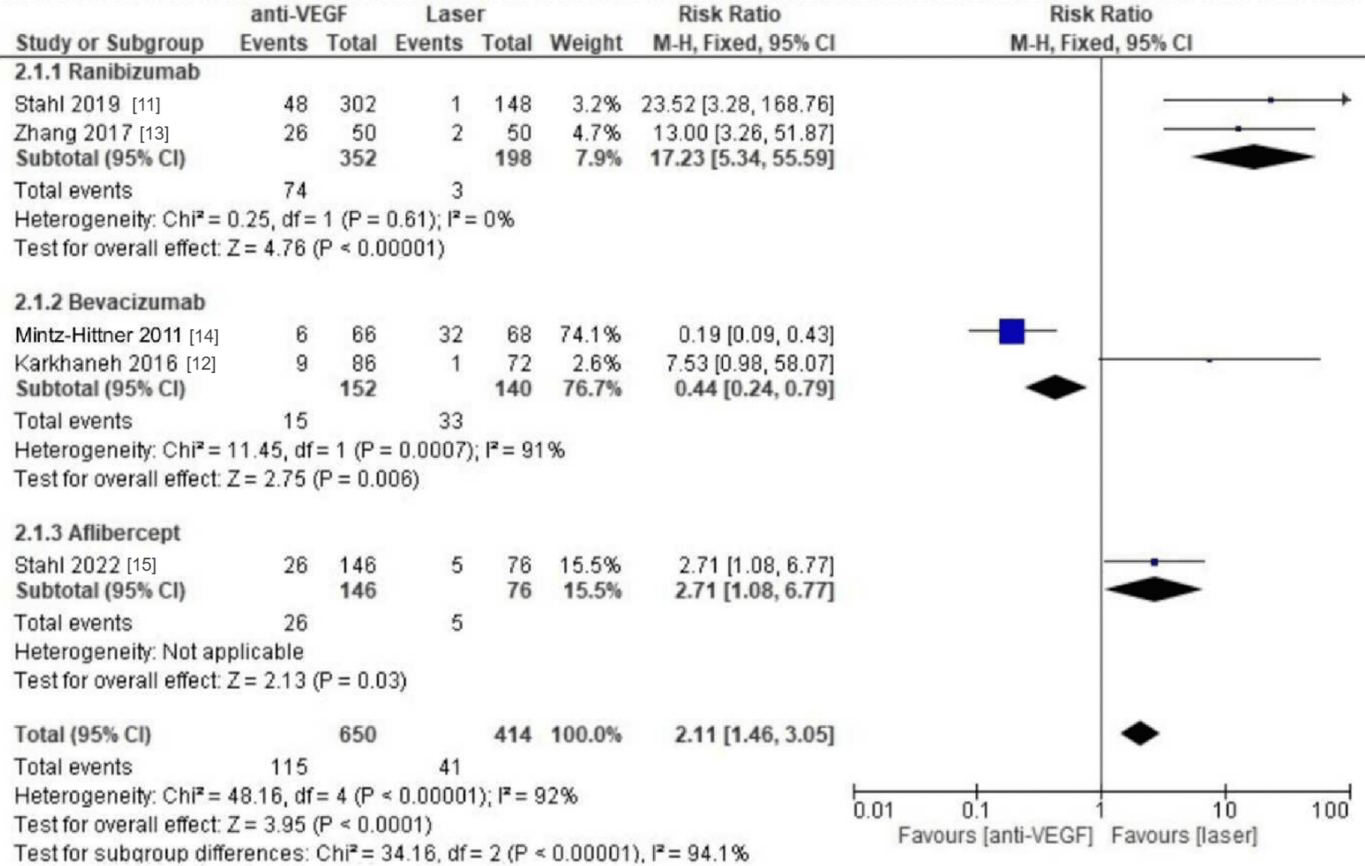
Forest plot for retreatment rates for laser therapy compared to anti-VEGF therapy regimens.

All five randomized controlled trials included in this review, comprising a total of 1064 eyes, reported retreatment rates for patients undergoing both anti-VEGF injections and laser therapy [11-15]. Our meta-analysis findings indicated that laser treatment was associated with a significantly lower risk of retreatment compared to anti-VEGF injections (RR = 2.11, 95% CI 1.46-3.05, p<0.0001, I^2 = 92%). Moreover, in subgroup analyses focusing on the three anti-VEGF medications, both the ranibizumab group (RR = 17.23, 95% CI 5.34-55.59, p<0.00001, I^2 = 0%) and the aflibercept group (RR = 2.71, 95% CI 1.08-6.77, p=0.03, I^2 = not applicable) demonstrated higher retreatment rates favoring laser treatment. Conversely, studies investigating bevacizumab indicated higher retreatment rates in patients receiving laser therapy compared to those receiving bevacizumab (RR = 0.44, 95% CI 0.24-0.79, p=0.006, I^2 = 91%), as illustrated in Figure 3.

Reactivation of Anti-VEGF vs. Laser Therapy

**Figure 4:**
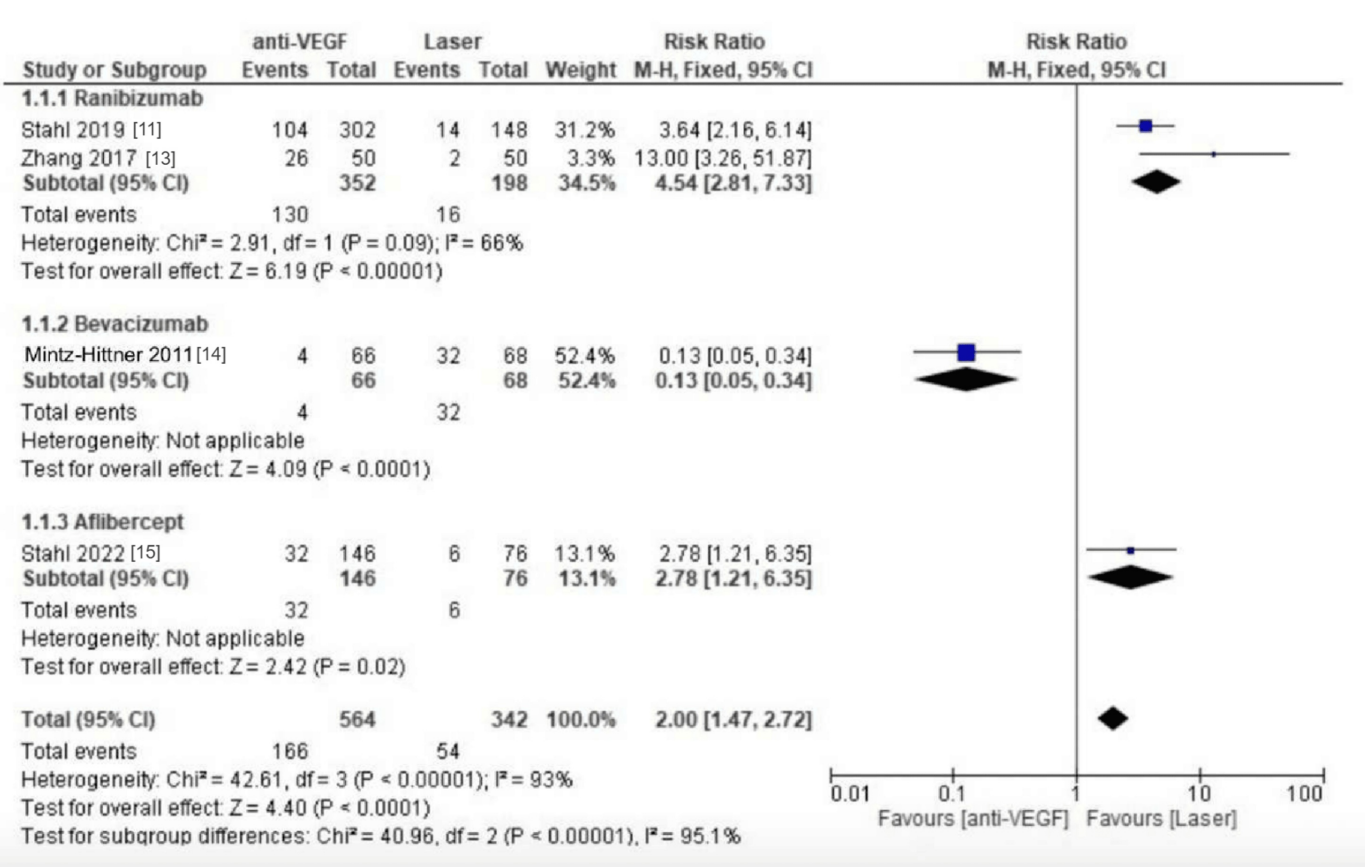
Forest plot for reactivation rates for laser therapy compared to anti-VEGF treatment regimens.

Out of the five RCTs we included in our study, only four trials reported reactivation rates. Overall, there was a significant difference in the reactivation rate between patients using anti-VEGF and laser therapy. Patients who underwent laser therapy had lower reactivation rates than the anti-VEGF groups in most trials (RR = 2.00, 95% CI: 1.47 to 2.72, p < 0.0001, I²=93%, Figure 4). In the subgroup analysis, both ranibizumab (RR = 4.54, 95% CI: 2.81 to 7.33, p=0.09, I²= 66%, Figure 4) and aflibercept (RR = 2.78, 95% CI: 1.21 to 6.35, I²=not applicable, Figure 4) showed higher reactivation rates, which favored laser treatment.

However, the trial that included bevacizumab (RR = 0.13, 95% CI: 0.05 to 0.34, I² = not applicable, Figure 4) reported lower reactivation with bevacizumab than laser therapy.

Discussion

Summary of Evidence

This study focused on assessing the risk of disease reactivation necessitating treatment in patients with ROP, drawing insights from five eligible RCTs. Among these RCTs, two investigated ranibizumab, two investigated bevacizumab, and one investigated aflibercept. In total, 1064 eyes diagnosed with ROP and treated with anti-VEGF agents were included, with 352 eyes receiving ranibizumab, 152 receiving bevacizumab, 146 receiving aflibercept, and a total of 414 undergoing laser therapy. Notably, patients undergoing laser therapy exhibited lower reactivation rates compared to those in the anti-VEGF groups across most trials.

Regarding retreatment rates, two studies utilizing ranibizumab reported 74 out of 352 eyes requiring retreatment, while 15 out of 152 eyes receiving bevacizumab and 26 out of 146 eyes receiving aflibercept required retreatment (Figure 2). Our findings indicate that laser treatment for ROP is linked to a reduced risk of retreatment compared to ranibizumab and aflibercept, consistent with a recent systematic review by Emer Chang et al. [16].

Conversely, bevacizumab demonstrated a reduction in both retreatment and reactivation rates when compared to laser therapy, supported by the BEAT-ROP trial, which established bevacizumab's superiority over laser in terms of a lower rate of reactivation requiring retreatment [14]. Additionally, the meta-analysis conducted in this review revealed that the anti-VEGF agent ranibizumab exhibited a higher retreatment rate compared to laser therapy, aligning with findings from the review by Jing Chen et al. [17].

Strengths and limitations

First, this review exclusively focused on randomized clinical trials, reflecting a high standard of evidence. Furthermore, it uniquely emphasized retreatment and reactivation rates following anti-VEGF treatment, distinguishing itself among systematic reviews by addressing this specific aspect. Nonetheless, the reliability of the data may have been compromised due to the limited number of randomized controlled trials assessing retreatment and reactivation rates of anti-VEGF agents for retinopathy of prematurity. Additionally, considerable heterogeneity was observed among the studies. A discrepancy arose in one of the five RCTs where the time between initial treatment and retreatment was not clearly reported, impacting the ability to draw precise conclusions regarding the optimal timing for each agent.

Future direction

Future research endeavors should place greater emphasis on investigating the long-term effects of anti-VEGF therapies on the progression of ROP, focusing on both the potential benefits and adverse outcomes that may manifest over time. It is noted that the majority of studies included in this review did not extensively explore the long-term implications of anti-VEGF treatment or the natural course of ROP. Hence, there is a clear imperative for studies dedicated to elucidating the extended outcomes of anti-VEGF therapy.

Additionally, there is a critical need to prioritize the prevention of reactivation in high-risk patients during the initial phases of therapy to mitigate potential complications in the future. Furthermore, there is a significant gap in understanding the severity of ROP, including factors such as zone and stage, in treated patients, which can provide valuable insights into the development of refractive errors. Regrettably, the limited availability of data on the incidence of refractive errors in patients prevented its inclusion in this review. Therefore, it is essential for future investigations to address this knowledge gap comprehensively.

## Conclusions

Our systematic review and meta-analysis scrutinize the efficacy of bevacizumab, ranibizumab, and aflibercept in contrast to laser therapy for treating retinopathy of prematurity (ROP), with a primary focus on the risk of disease reactivation necessitating retreatment. The findings reveal that laser therapy outperforms both ranibizumab and aflibercept, demonstrating a reduced risk of disease reactivation requiring retreatment. However, investigations comparing bevacizumab to laser therapy suggest that bevacizumab exhibits lower rates of retreatment and reactivation compared to the laser treatment groups.
